# Advances in Imaging and Diagnosis of Emphysematous Cholecystitis

**DOI:** 10.3390/healthcare14050617

**Published:** 2026-02-28

**Authors:** Kathleen H. Miao, Julia H. Miao, Sonam Rosberger, Abraham H. Dachman, Bachir Taouli, Sara C. Lewis

**Affiliations:** 1 Department of Diagnostic, Molecular, and Interventional Radiology, Icahn School of Medicine at Mount Sinai, New York, NY 10029, USA; 2 Department of Radiology, University of Chicago Medicine, Chicago, IL 60637, USA

**Keywords:** emphysematous cholecystitis, acute cholecystitis, radiology, radiograph, computed tomography, magnetic resonance imaging, dual-energy computed tomography, photon-counting computed tomography, artificial intelligence

## Abstract

Emphysematous cholecystitis is a rare but severe variant of acute cholecystitis characterized by gas-forming organisms within the gallbladder wall or lumen. It progresses rapidly and carries substantial mortality, making early and accurate recognition essential. Although its pathogenesis involves gallbladder wall ischemia with superimposed infection by gas-producing bacteria—most commonly *Clostridium* species—the clinical presentation is often nonspecific, particularly in patients with diabetes mellitus or immunosuppression. Imaging therefore serves as the cornerstone of diagnosis. Abdominal radiographs may demonstrate intraluminal or intramural gas, while ultrasound can reveal echogenic foci with reverberation artifacts, though overlying bowel gas and diagnostic mimics may limit sensitivity. Computed tomography remains the most accurate modality, precisely delineating gas within the gallbladder wall, lumen, or adjacent tissues and facilitating urgent surgical or percutaneous intervention. Magnetic resonance imaging offers complementary soft tissue characterization when computed tomography is contraindicated. This review synthesizes traditional imaging findings and emerging diagnostic innovations by critically comparing modality-specific strengths, limitations, and pitfalls. Dual-energy and photon-counting computed tomography enhance tissue contrast and gas conspicuity, while artificial intelligence-assisted image analysis enables earlier detection and expedited triage in emergency settings. By integrating evolving technologies with established radiologic principles, this article provides a forward-looking framework for improving diagnostic precision and ultimately enhancing outcomes for patients with emphysematous cholecystitis.

## 1. Introduction

Emphysematous cholecystitis (EC) can be a severe and potentially fatal form of acute necrotizing cholecystitis characterized by the presence of gas within the gallbladder wall, lumen, or pericholecystic tissues, produced by gas-forming microorganisms. EC can occur in the presence or absence of gallstones and is associated with a higher risk of complications, including perforation, gangrene, sepsis, and higher mortality rates. The disease predominantly affects elderly, diabetic, and immunocompromised patients, in whom vascular insufficiency and impaired immune defense may contribute to its pathogenesis [1].

The underlying mechanism of EC involves ischemic injury to the gallbladder wall, predisposing the organ to infection by anaerobic or facultative anaerobic bacteria capable of gas production, such as *Clostridium perfringens*, *Escherichia coli*, and *Klebsiella* species [2,3]. This ischemia–infection interplay leads to the rapid accumulation of gas in the gallbladder tissues, often progressing faster than typical acute calculus cholecystitis and necessitating urgent medical or surgical intervention. Thus, although EC accounts for approximately 1% of all acute cholecystitis cases, EC is associated with markedly higher rates of morbidity and mortality [1], making it especially important for early, accurate diagnosis.

Given the nonspecific clinical presentation and the rapid progression of the disease, early diagnosis is essential to improving patient outcomes. Imaging plays an important role in the identification and characterization of EC. Abdominal radiography may reveal gas in the right upper quadrant (RUQ), although sensitivity is limited. Ultrasonography, commonly used as the first-line modality for RUQ pain, may show echogenic foci with dirty shadowing or reverberation artifacts consistent with gas; however, it is often limited by bowel gas or patient body habitus. Computed tomography (CT) has emerged as the most sensitive and specific imaging modality, providing detailed visualization of intramural and pericholecystic gas, gallbladder wall thickening, and associated complications such as perforation or abscess formation [1]. Magnetic resonance imaging (MRI) provides excellent soft tissue contrast and can be particularly valuable in patients where radiation exposure is a concern. Advanced sequences such as diffusion-weighted imaging (DWI) and MR cholangiopancreatography (MRCP) enhance the detection of gallbladder wall inflammation, edema, and biliary tract involvement.

This article reviews the pathophysiology and imaging characteristics of EC, highlighting the critical role of imaging modalities in facilitating early and accurate diagnosis. Advances in imaging, enhanced awareness, and timely imaging can expedite intervention, reduce morbidity and mortality, and ultimately improve clinical outcomes in affected patients.

A review was conducted to comprehensively evaluate the imaging features, diagnostic performance, and evolving radiologic techniques related to emphysematous cholecystitis. The review methodology was designed to ensure transparency, reproducibility, and methodological rigor.

Electronic searches were performed in PubMed and Scopus, which were selected for their robust indexing of biomedical and radiologic literature. Searches were conducted for studies published between 2002 and 2025 to reflect evolving contemporary imaging practices and technological advancements.

The search strategy employed Boolean operators and controlled vocabulary where applicable, combining keywords such as “emphysematous cholecystitis,” “gas-forming cholecystitis,” “computed tomography,” “ultrasound,” “magnetic resonance imaging,” and “radiologic diagnosis.” Only English-language publications were included. Eligible studies comprised original research articles, clinical trials, observational studies, meta-analyses, and systematic reviews focusing on radiologic evaluation or imaging-based diagnosis. Non-peer-reviewed sources were excluded.

Study selection was performed in a two-stage process. Titles and abstracts were initially screened for relevance, followed by full-text review to confirm eligibility based on predefined inclusion and exclusion criteria. Discrepancies in study selection were resolved through consensus review. The reference lists of included articles were manually screened to identify additional relevant studies not captured in the primary search. While formal quantitative meta-analysis was not feasible due to heterogeneity in study design and outcomes, qualitative synthesis was performed, and studies were assessed for methodological quality and potential sources of bias.

The initial database search yielded 412 records, of which 96 duplicates were removed. Following title and abstract screening of 316 unique articles, 247 studies were excluded due to lack of relevance to radiologic evaluation, non–imaging-focused content, or publication type. Full-text review was performed on 79 articles, of which 38 were excluded for insufficient imaging detail, limited methodological quality, or redundancy. Ultimately, 41 articles met the inclusion criteria and were incorporated into the qualitative synthesis and review.

This structured approach allowed for a comprehensive synthesis of established imaging paradigms and emerging diagnostic innovations, reinforcing the central role of radiology in the early detection and management of emphysematous cholecystitis.

## 2. Clinical Presentation

EC represents a severe manifestation of acute cholecystitis with a unique underlying pathophysiology distinct from the more common calculous form. The central mechanism involves ischemic insult to the gallbladder wall, followed by secondary infection with gas-forming organisms. The gallbladder’s blood supply, derived from the cystic artery—a terminal branch of the right hepatic artery—is susceptible to compromise in patients with systemic vascular disease, especially those with diabetes mellitus, which is an established risk factor for EC [1].

Ischemia results in mucosal necrosis and a breakdown of the mucosal barrier, allowing translocation and proliferation of enteric bacteria, particularly anaerobic and facultative anaerobic organisms. The most commonly implicated pathogens include *Clostridium perfringens*, *Klebsiella pneumoniae*, and *Escherichia coli* species, all of which are capable of producing hydrogen and nitrogen gas as metabolic byproducts [3]. *Clostridium perfringens* has been isolated in a significant number of EC cases and is known for its ability to cause rapid tissue destruction and gas gangrene in soft tissues, suggesting a similar pathogenic mechanism in the gallbladder [3].

The presence of gas within the gallbladder wall or lumen is an important finding for EC. The gas dissects through necrotic tissue planes, producing intramural emphysema and sometimes extending into the pericholecystic tissues or biliary tree (pneumobilia), particularly in advanced cases. Inflammatory cytokine release, neutrophilic infiltration, and microvascular thrombosis further propagate the ischemic cycle, contributing to gallbladder gangrene and perforation if left untreated [4].

Patients with diabetes are at heightened risk for emphysematous cholecystitis due to a confluence of pathophysiologic factors. Concurrent microvascular disease compromises perfusion, predisposing the gallbladder to hypoperfusion most commonly due to cystic artery insufficiency, initiates a cascade of tissue necrosis and secondary infection by gas-forming organisms such as *Escherichia coli* and *Klebsiella* [1]. This fosters anaerobic bacterial proliferation and increases susceptibility to complications including gangrene, perforation, and pericholecystic abscess formation [1]. Reduced arterial perfusion facilitates anaerobic growth of organisms such as *Clostridium* [5,6,7]. Patients with advanced atherosclerosis or peripheral vascular disease therefore have a higher likelihood of developing ischemic cholecystitis, which can evolve into EC.

Thus, EC develops from a confluence of the above mechanisms, leading to the radiological and pathological findings of the disease and underscoring the need for early diagnosis and prompt intervention to prevent severe complications.

The clinical presentation of EC often overlaps with that of typical acute cholecystitis, which can make early recognition challenging. However, EC tends to progress into a more rapidly deteriorating course and is associated with higher morbidity and mortality [2]. The classic symptoms include RUQ abdominal pain, fever, nausea, vomiting, and leukocytosis, but these may be subtle or absent, especially in elderly, diabetic, or immunocompromised patients [1,3].

Pain is typically severe and localized to the RUQ, often radiating to the right shoulder or back due to stimulation of the right phrenic nerve and intercostal nerves (T5-T11 dermatomes) [5]. Fever is common. A positive sonographic Murphy sign—focal tenderness during inspiration with gallbladder palpation—is a helpful diagnostic feature for patients with acute calculous cholecystitis but is less prevalent for those with acute gangrenous cholecystitis or EC for which its absence may suggest gangrene [5].

Patients with EC are often hemodynamically unstable on presentation, with hypotension and tachycardia suggesting systemic inflammatory response or early sepsis. Approximately up to 25% of patients may progress to septic shock, which can be lethal [6].

The occurrence of EC correlates with a rise in patient age with the majority of cases (>50%) occurring in the age group of 50 years and older, especially those in the sixth to seventh decade. Its prevalence is three times higher in men compared to women [1]. In elderly or diabetic patients with unexplained sepsis or abdominal symptoms, EC should remain high on the differential diagnosis, even in the absence of overt peritoneal signs or fever.

Furthermore, patients with impaired immunity due to corticosteroid therapy, chemotherapy, solid-organ transplantation, human immunodeficiency virus (HIV) infection, or cancer diminish the host’s ability to clear biliary pathogens. This immunologic deficit not only raises the risk of severe gallbladder infections but also allows opportunistic and gas-forming organisms to proliferate unchecked. Immunosuppressed patients often present with advanced or atypical manifestations, contributing to higher complication rates of EC.

Patients with end-stage renal disease have chronic immune dysfunction, uremia-associated metabolic derangements, and vascular calcifications that impair local perfusion. Hemodialysis further exposes patients to recurrent bacteremia and hemodynamic fluctuations. Consequently, advanced kidney disease and dialysis are comorbidities in EC cohorts, with increased risk of both development and poor outcomes.

Patients in intensive care settings or those with systemic shock, sepsis, or trauma are vulnerable to ischemic injury of the gallbladder [1]. Low-flow states reduce cystic artery perfusion, and prolonged hypotension or vasopressor use worsens mucosal hypoperfusion. In this setting, even transient colonization of the gallbladder can progress rapidly to EC. Critical illness is thus a recognized risk factor for EC.

Laboratory findings in cholecystitis are nonspecific but typically reveal evidence of systemic inflammation and biliary obstruction [8,9,10]. Common abnormalities include: Leukocytosis (often marked), elevated C-reactive protein, mildly elevated liver function tests (particularly alkaline phosphatase and gamma-glutamyl transferase) and hyperbilirubinemia [8,9,10].

Bile cultures with emphysematous cholecystitis demonstrated positive cultures, growing *Clostridium* species, *Clostridium welchii*, *Escherichia coli,* and *Bacteroides fragilis* [7,11]. These findings highlight the polymicrobial and anaerobic nature of the infection, supporting the need for broad-spectrum antimicrobial coverage.

Due to the nonspecific nature of these clinical and laboratory features, early imaging is essential for diagnosis, as physical findings alone are often insufficient to differentiate EC from other forms of cholecystitis or intra-abdominal infection.

## 3. Radiological Work-Up

### 3.1. Radiographs

Plain abdominal radiography, though less sensitive than ultrasound or CT, can serve as an initial imaging modality in the evaluation of patients presenting with abdominal pain, particularly in emergency or resource-limited settings. Radiographs may demonstrate hallmark features of EC when a significant amount of gas is present within the gallbladder lumen, wall, or surrounding tissues [12,13]. Although not diagnostic on its own, radiography can provide early clues that prompt further, more definitive imaging.

The classic findings on plain radiographs include emphysematous mural gas within the gallbladder wall, intraluminal gas, and pericholecystic gas, which may appear as curvilinear lucencies outlining the gallbladder or as mottled, air-fluid levels in the RUQ [13]. Free intraperitoneal air may also be seen if perforation has occurred. Patient positioning can aid in EC diagnosis because gas redistributes to the nondependent portion and may rise or layer, demonstrating mobility on decubitus or upright imaging.

A supine abdominal radiograph may show crescent-shaped or linear lucencies corresponding to gas within the gallbladder wall. In some instances, the gallbladder outline becomes visible due to the presence of gas surrounding it. Erect radiographs may also reveal air-fluid levels in the RUQ if intraluminal gas has accumulated [13,14]. The presence of gas in these locations seen on imaging may have been introduced during procedures or surgeries such as endoscopic retrograde cholangiography, post sphincterotomy or choledocho-enteric anastomosis, so knowledge of a patient’s clinical and surgical history is essential.

Despite these potential findings, radiography has limited sensitivity and specificity in diagnosing EC, particularly in early stages or when gas volumes are small [15]. Furthermore, bowel gas, obesity, and overlying soft tissues can obscure visualization of the gallbladder, further limiting diagnostic accuracy.

Nonetheless, radiographs may offer rapid, preliminary evidence of a gas-forming infection and should not be overlooked in acutely ill patients with nonspecific abdominal pain, especially when CT or ultrasound is not immediately available. In the appropriate clinical setting, particularly in diabetic or immunocompromised patients with signs of sepsis, radiographic identification of RUQ gas should raise strong suspicion for EC and prompt urgent further imaging with CT, which remains the imaging reference standard [15].

### 3.2. Ultrasound

Ultrasound is often the first-line imaging modality for evaluating RUQ abdominal pain and suspected acute cholecystitis due to its wide availability, lack of ionizing radiation, and high sensitivity for gallstones and gallbladder inflammation [13]. In the context of EC, however, ultrasound presents unique challenges and specific diagnostic findings that radiologists and clinicians must recognize to avoid delayed diagnosis.

**Figure 1:**
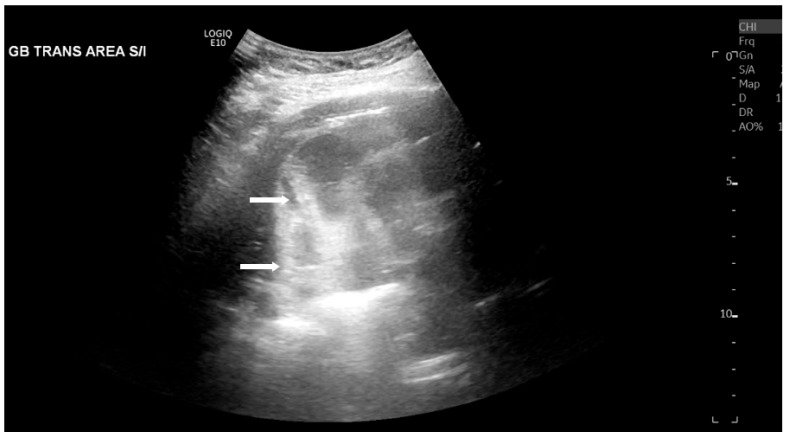
Right upper quadrant ultrasound of a 63-year-old male diabetic patient with emphysematous cholecystitis. Posterior dirty shadowing (white arrows) due to gas within the gallbladder wall were demonstrated.

**Figure 2:**
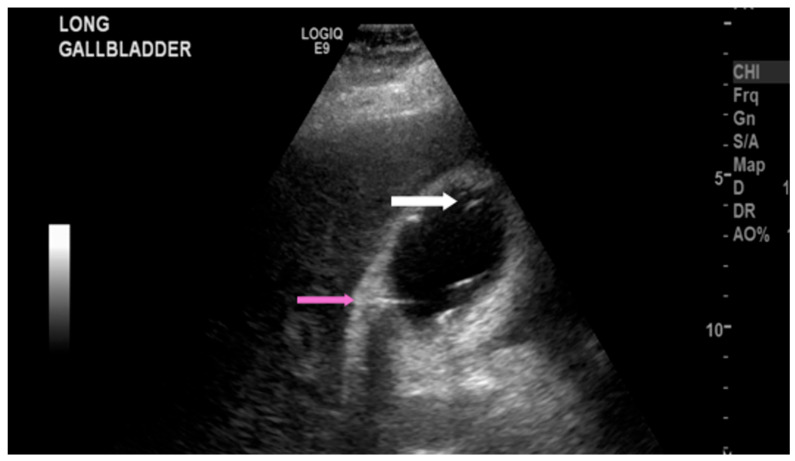
Right upper quadrant ultrasound of a 52-year-old female human immunodeficiency virus patient with emphysematous cholecystitis. Foci of air in the gallbladder lumen (white arrow) and intramural air (pink arrow) were demonstrated.

The most common sonographic finding of EC is the presence of gas within the gallbladder wall or lumen, which appears as highly echogenic foci accompanied by posterior dirty shadowing or reverberation artifacts, such as ring-down artifact, shown in Figure 1 and Figure 2. These artifacts are due to reflection and scattering of ultrasound waves by intraluminal gas. When the hyperechoic foci layer in the non-dependent portion of the gallbladder is present with low-level posterior shadowing, the appearance has been termed “the champagne sign” [16]. Furthermore, with changes in patient positioning, the gas may demonstrate mobility or layering on decubitus or upright imaging, aiding EC diagnosis. However, potential mimics of the visualized gas include gas secondary to recent instrumentation, biliary–enteric anastomoses, or sphincterotomy, which are important to consider clinically.

**Figure 3:**
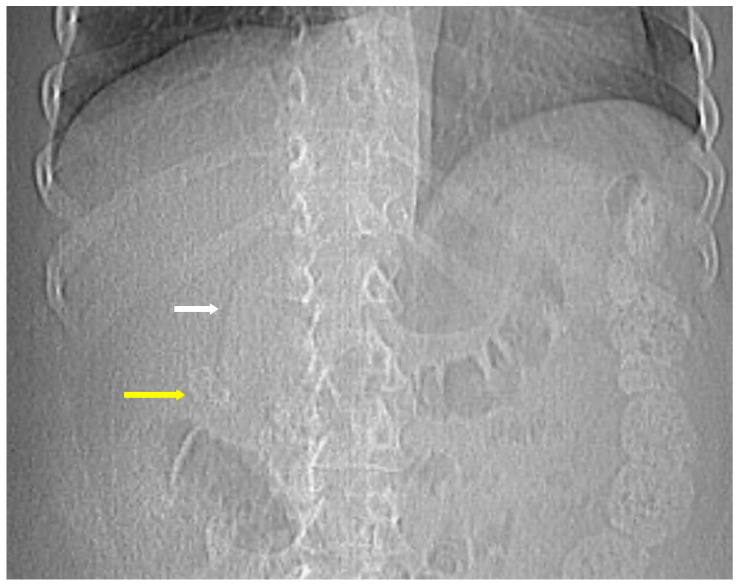
The initial scout image of a computed tomography abdomen and pelvis for a 49-year-old female diabetic patient. Curvilinear lucencies (white arrow) outlining the gallbladder due to emphysematous mural gas within the gallbladder wall and cholelithiasis (yellow arrow) were demonstrated.

Additionally, the gallbladder wall may appear thickened (>3 mm) or irregular due to ischemic changes, inflammation, or gas dissection into the wall layers [13], as seen in Figure 3. Pericholecystic fluid and sludge may also be seen and are common but nonspecific findings.

Despite these characteristic features, ultrasound has significant limitations in detecting EC, particularly in its early stages or when gas volumes are small. Moreover, overlying bowel gas and body habitus may obscure the acoustic window, complicating interpretation. Importantly, false-negative results may occur when the gas is confined to the gallbladder wall or in small amounts [14]. Conversely, false positives may occur if intraluminal echogenic foci are misinterpreted as gallstones with reverberation, highlighting the need for sonographic correlation with clinical findings and further imaging.

Despite its utility as an initial imaging modality, ultrasound evaluation of emphysematous cholecystitis is subject to several important diagnostic pitfalls that can complicate accurate interpretation. They include interobserver variability and dependence on operator expertise. Furthermore, intramural or intraluminal gas may produce echogenic foci with posterior dirty shadowing or reverberation artifacts that can mimic other gallbladder pathologies. In particular, differentiation from porcelain gallbladder is critical, as mural calcification produces dense echogenic walls with clean posterior acoustic shadowing rather than the irregular, dirty shadowing characteristic of gas [7]. Similarly, comet-tail artifacts, commonly seen in adenomyomatosis, arise from cholesterol crystals within Rokitansky–Aschoff sinuses and are typically associated with stable wall thickening rather than acute inflammatory changes. Aerobilia represents another potential mimic, as gas within the biliary tree may project over the gallbladder region; however, aerobilia typically demonstrates central branching echogenicity within the liver and changes with patient positioning, in contrast to fixed intramural gas in emphysematous cholecystitis [13]. Awareness of these mimics and careful correlation with clinical findings and complementary imaging—particularly CT—are essential to avoid misdiagnosis and delays in definitive management.

Given these limitations, ultrasound remains a valuable initial tool, but in cases of suspected EC, especially in diabetic or septic patients with atypical findings, there should be a low threshold to perform CT to confirm the diagnosis, assess the extent of disease, and help guide management.

### 3.3. CT

CT is a highly sensitive imaging modality for diagnosing EC [17] and is considered particularly useful when ultrasound findings are inconclusive or limited by patient body habitus or overlying bowel gas. CT provides rapid, high-resolution visualization of the gallbladder and surrounding structures, making it indispensable for detecting gas in atypical or early-stage presentations.

**Figure 4:**
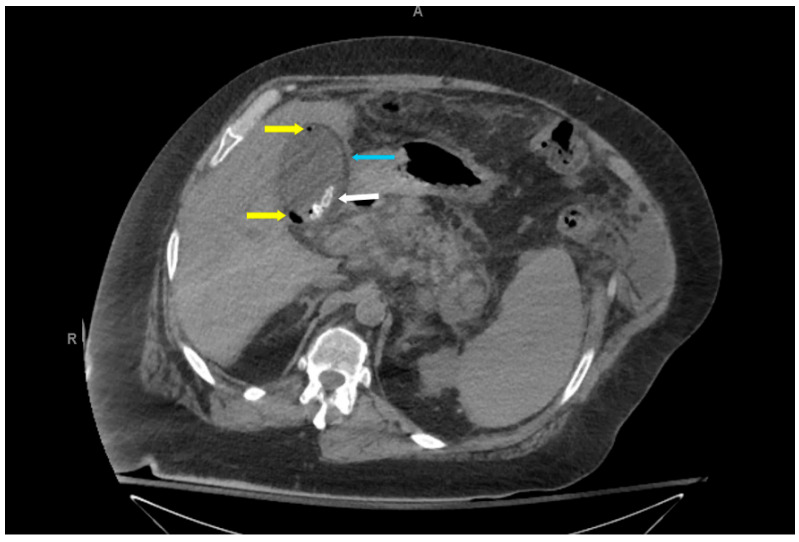
Axial non-contrast computed tomography abdomen (slice thickness 3 mm; soft tissue window, WW 350, WL 40) of a 61-year-old diabetic male patient with emphysematous cholecystitis presenting with right upper quadrant pain. Cholelithiasis (white arrow), foci of air in the gallbladder lumen and wall (yellow arrows), and gallbladder wall edema (blue arrow) were incidentally noted.

**Figure 5:**
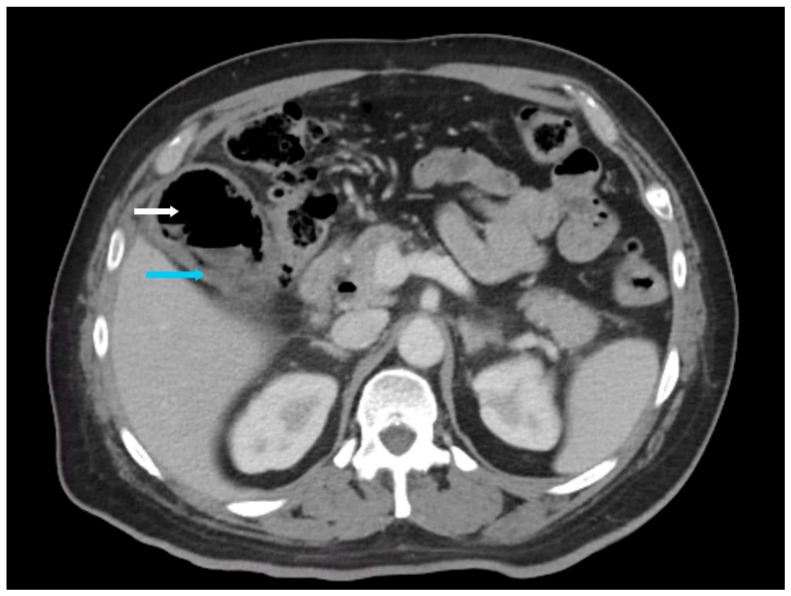
Axial contrast-enhanced computed tomography abdomen in the arterial phase (slice thickness 3 mm; soft tissue window, WW 350, WL 40) of a 39-year-old male diabetic patient with emphysematous cholecystitis presenting with abdominal pain. Extensive air in the gallbladder lumen (white arrow) and gallbladder wall edema (blue arrow) were incidentally noted.

The hallmark CT findings of EC include the presence of gas within the gallbladder wall, lumen, or pericholecystic tissues. Gas may appear as curvilinear, punctate, or mottled foci along the gallbladder wall, as seen in Figure 3, Figure 4 and Figure 5. Intraluminal gas can create air-fluid levels or completely fill the gallbladder, while gas in adjacent tissues may signify transmural necrosis or perforation [7]. CT can also identify complications such as pericholecystic abscess, emphysematous spread to adjacent organs (e.g., liver or duodenum), or intraperitoneal free air in the case of perforation and rupture [18].

In addition to identifying gas, contrast-enhanced CT allows for detailed assessment of gallbladder wall thickening, stratification, edema, and vascular compromise, which may suggest progression to gangrenous cholecystitis [19]. CT is particularly effective in detecting subtle or early emphysematous changes. Moreover, noncontrast CT may be sufficient for diagnosis in some cases, as gas is readily visible without intravenous contrast. However, contrast-enhanced CT can provide additional information regarding gallbladder perfusion and the presence of adjacent inflammation or abscess formation, which may influence management decisions.

CT plays a crucial role not only in diagnosing EC but also in guiding treatment strategy. Patients demonstrating extensive gallbladder necrosis, perforation, or signs of peritonitis typically require urgent surgical cholecystectomy to prevent severe complications [20]. Conversely, critically ill patients, very elderly patients, or those with significant comorbidities who are poor surgical candidates may benefit from percutaneous cholecystostomy as a temporizing or definitive measure to control infection and inflammation [21].

Thus, CT offers unparalleled diagnostic accuracy for EC and should be promptly performed in any patient with clinical suspicion, especially when initial radiographs or ultrasound are inconclusive. Its ability to delineate gas distribution, assess complications, and guide management makes it an essential tool in improving outcomes in this high-risk condition.

### 3.4. MRI

MRI is typically not the first-line modality for evaluating EC due to its longer acquisition times and sensitivity to patient motion, which can pose challenges in acute settings. However, MRI is a valuable alternative for those such as pregnant or pediatric patients where CT is relatively contraindicated, and is increasingly used in clinical practice. Furthermore, it offers superior soft tissue contrast. Although MRI is not the primary emergency modality in unstable patients with suspected cholecystitis in the acute setting, it can be used for problem solving following stabilization.

**Figure 6:**
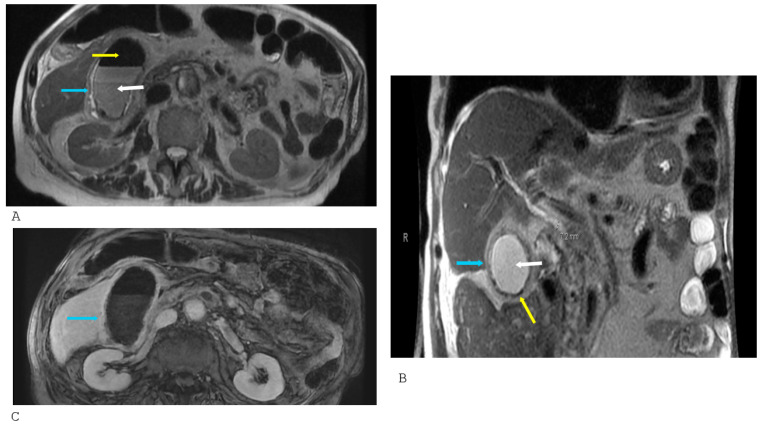
Magnetic resonance imaging of a 53-year-old female diabetic patient with emphysematous cholecystitis. (**A**,**B**): Axial T2 and coronal T2 sequences demonstrate gallbladder sludge (white arrow), intraluminal gas and intramural gas signified by the gas-related signal voids (yellow arrows), and gallbladder wall edema (blue arrow); (**C**): Post-contrast axial sequence demonstrates mucosal hyperenhancement (blue arrow) of the gallbladder wall. The lack of enhancement of the gas-related signal voids (yellow arrows) and signal void on all sequences distinguishes them from susceptibility and flow-related artifacts, which would enhance.

MRCP is increasingly utilized to assess suspected biliary duct obstructions, and cases of EC may be incidentally detected during these evaluations. MRCP findings indicative of EC include increased T2 signal intensity reflecting gallbladder wall edema, signal voids indicating the presence of gas, and blooming artifacts on gradient echo sequences due to gas presence [22]. Advanced sequences, including DWI, further improve sensitivity for detecting gallbladder wall inflammation, edema, and associated biliary tract involvement. Figure 6 illustrates MRI findings in a diabetic patient with EC.

Beyond conventional MRI sequences, multiparametric imaging can enhance the characterization of emphysematous cholecystitis. T1-weighted imaging may demonstrate hypointense gallbladder wall regions corresponding to edema or necrosis, while T2-weighted sequences accentuate wall thickening and pericholecystic fluid collections. Gradient echo sequences are particularly sensitive to small foci of intramural gas, which appear as signal voids and can be distinguished from adjacent vascular structures or bile. The combination of T2 hyperintensity with corresponding restricted diffusion on DWI sequences provides a non-invasive biomarker of active inflammation, allowing differentiation of emphysematous changes from chronic wall thickening or gallbladder sludge. Additionally, post-contrast T1-weighted imaging can delineate areas of mucosal enhancement versus non-enhancing necrotic segments, thereby aiding in the early detection of gangrenous transformation and informing surgical planning [22].

MRI also serves a valuable role in evaluating associated complications and comorbid biliary pathology. Emphysematous cholecystitis may coexist with choledocholithiasis, biliary strictures, or portal venous gas, all of which can be accurately assessed with MRCP and three-dimensional reconstructions. MRCP facilitates visualization of the entire biliary tree without ionizing radiation, allowing identification of ductal obstruction or secondary infection. In addition, MRI can help distinguish intramural gas from mimics such as porcelain gallbladder or intraluminal sludge, especially when CT findings are equivocal. While not a first-line modality in unstable patients during emergencies, MRI provides a comprehensive, non-invasive assessment for EC in stable patients, expanding the clinician’s ability to plan tailored interventions and anticipate complications [17].

### 3.5. Imaging and Clinical Considerations

For patients with suspected or confirmed emphysematous cholecystitis, CT is often prioritized as the first-line imaging modality due to its superior sensitivity for detecting intramural, intraluminal, and pericholecystic gas—the defining diagnostic feature of EC—and its ability to assess disease extent, complications, and alternative diagnoses in acutely ill patients. CT rapidly delineates gallbladder wall gas, perforation, abscess formation, and vascular compromise, making it the most clinically actionable modality in unstable or high-risk populations such as older adults, or those presenting with sepsis or atypical symptoms [1,2,3]. In contrast, radiographs may be an initial imaging test for abdominal pain and incidentally detect gas in the gallbladder. Furthermore, while US is often the initial imaging test in suspected uncomplicated acute cholecystitis due to accessibility and lack of ionizing radiation, the sensitivity for EC is limited by bowel gas, patient body habitus, and operator dependence, and gas within the gallbladder wall may obscure diagnostic landmarks or mimic shadowing calculi [7]. As such, US is best reserved for initial evaluation in hemodynamically stable patients or when CT is contraindicated, with a low threshold to escalate to CT if EC is clinically suspected.

MRI and MRCP are not routinely indicated in the acute diagnostic workflow of EC but may serve a complementary role in select clinical scenarios, such as when biliary obstruction, choledocholithiasis, or malignancy is suspected following stabilization. Therefore, a pragmatic, decision-based imaging strategy prioritizes emergent CT for definitive diagnosis and surgical planning, with US and MRI functioning as adjunctive modalities tailored to patient stability, comorbidities, and diagnostic uncertainty. This improves diagnostic efficiency and clinical applicability in the management of this high-mortality condition.

## 4. Treatment and Management

EC represents a surgical emergency due to its rapidly deteriorating course, risk of rapid progression to gangrene, perforation, sepsis, and mortality [1]. Prompt recognition on imaging and aggressive management, often requiring both medical stabilization and surgical intervention, are essential to improving outcomes. Upon clinical suspicion or imaging diagnosis of EC, hemodynamic stabilization is the first priority. Initial management includes (1) intravenous fluid resuscitation to address hypovolemia or septic shock; and (2) broad-spectrum intravenous antibiotics covering anaerobic and Gram-negative organisms, particularly *Clostridium* spp., *Klebsiella pneumoniae*, and *Escherichia coli* [3]. Common empiric regimens include piperacillin–tazobactam, meropenem, or a combination of a third-generation cephalosporin with metronidazole. Furthermore, institutional variability or resistance patterns affect antibiotic regimens. Blood and bile cultures should be obtained prior to antibiotic administration, if possible, to allow for targeted therapy. Follow-up imaging after antibiotic administration can be used for monitoring inadequate clinical improvement or concern for further complications.

Urgent cholecystectomy remains the definitive treatment for most patients with EC [2]. Laparoscopic cholecystectomy is feasible in selected stable patients; however, due to extensive inflammation, necrosis, and friability of the gallbladder wall, the procedure often requires conversion to an open approach. Early surgical intervention is associated with improved survival and reduced complications. In high-risk surgical candidates, particularly elderly, diabetic, or critically ill patients, percutaneous cholecystostomy under ultrasound or CT guidance may serve as a temporizing or definitive measure [11]. This image-guided drainage procedure decompresses the inflamed gallbladder and allows bile sampling for culture, often leading to clinical improvement [11]. Imaging plays an important role in not only detection but also treatment and clinical monitoring if complications after cholecystectomy, such as bile duct injury, bile leak, abscesses, and bleeding.

## 5. Prognosis

EC mortality rates are higher than those of uncomplicated acute cholecystitis [1]. Patients frequently experience complications such as gallbladder gangrene, perforation, peritonitis, and septic shock, each of which further increases the likelihood of death. Delay in presentation is strongly correlated with adverse outcomes, as gallbladder necrosis and perforation can occur within 24–48 h of symptom onset.

Post-procedural care includes continued antibiotics, serial laboratory monitoring, and repeat imaging if clinical deterioration occurs. Management of underlying comorbidities is also important, as these factors contribute to poor clinical outcomes and risk of recurrence. Patients who undergo percutaneous cholecystostomy should be reassessed for interval cholecystectomy once stabilized, as recurrent episodes of EC or cholecystitis may occur if the diseased gallbladder is left in situ.

EC continues to carry a high risk of complications. Predictors of poor outcomes associated with increased mortality of patients with complicated cholecystitis include patients with comorbidities, increased delay time prior to hospital admission, and low white blood cell count [22,23], such as patients with HIV and cancer patients on chemotherapy.

In addition, renal failure and vascular disease have been shown to worsen prognosis due to impaired tissue perfusion and reduced physiological reserve [1]. Patients with end-stage renal disease on dialysis are particularly vulnerable. Similarly, these patients often present in hemodynamically unstable states, compounding perioperative risk.

Thus, laboratory and clinical predictors of poor outcomes include hypotension at admission, acute kidney injury, and leukopenia in immunocompromised patients. Imaging findings such as gallbladder wall perforation, pericholecystic abscess, or pneumoperitoneum indicate advanced disease and correlate with increased mortality. Patients requiring vasopressors, mechanical ventilation, or those who develop multiorgan dysfunction in the setting of septic shock have worse outcomes.

Ultimately, prognosis depends on age, comorbidities, immunologic status, disease severity at presentation, and timeliness of intervention. Early recognition, aggressive resuscitation, broad-spectrum antimicrobial coverage targeting anaerobic and gas-forming organisms, and prompt surgical or percutaneous drainage are important for improving chances of survival. Imaging plays an important role in detection, clinical monitoring, and prognosis.

## 6. Discussion

In recent years, technological advancements have significantly improved the diagnosis, monitoring, and treatment strategies for EC. Given the high mortality and diagnostic challenge associated with EC, there is a growing emphasis on enhancing imaging sensitivity and applying artificial intelligence (AI).

### 6.1. Dual-Energy CT and Photo-Counting CT

Dual-energy CT (DECT) represents a significant advancement over conventional CT in the evaluation of gallbladder pathology, particularly in conditions such as EC. By utilizing two different energy levels, high and low, DECT exploits the differentiation of various tissue types, thereby improving soft tissue contrast and facilitating characterization of the organs and tissues based on the differential of their attenuation [24]. This enables material decomposition, virtual monoenergetic image reconstruction, and iodine quantification that surpass conventional single-energy CT in tissue characterization and contrast conspicuity.

In a study of patients with cholecystitis from 2018 to 2021 led by Huda et al., the diagnosis of acute cholecystitis using DECT was associated with higher sensitivity compared to conventional CT in detecting gallbladder fossa hyperemia, gangrene, heterogeneous wall enhancement, and cholecystitis [25]. Overall, DECT had a higher sensitivity of 89.5% versus conventional CT, which had a sensitivity of 70.2% [25]. Thus, DECT is particularly beneficial in identifying subtle intramural gas and ischemic changes that are characteristic of necrotic or inflamed gallbladders with cholecystitis.

In another retrospective study of cholecystitis patients from 2017–2021 led by Nevo et al., DECT was found to aid in better visualization of gallbladder wall integrity, with a sensitivity of 93.8%, and pericholecystic hepatic enhancement, with a sensitivity of 96.9%, which had a strong correlation with positive bile cultures in cholecystitis patients, affecting clinical management and interventional decision making [26].

In the context of EC, where rapid and accurate diagnosis is crucial due to the risk of severe complications, DECT can provide more detailed visualization of gas patterns within the gallbladder wall. This enhanced imaging allows for the detection of early ischemic changes that might be missed on conventional CT, potentially facilitating earlier intervention and improving patient outcomes. The ability of DECT to better characterize these critical features, using both high and low energy levels, underscores its promise as a valuable tool in the diagnostic arsenal for complex gallbladder conditions, improving the detection of acute cholecystitis [27].

Photon-counting computed tomography (PCCT) is an emerging imaging technology that enhances diagnostic capability through superior spatial resolution, reduced image noise, and energy-resolved data acquisition [28]. PCCT uses semiconductor detectors that directly convert individual X-ray photons into electrical signals while simultaneously measuring their energy. This enables material decomposition and the generation of iodine maps, which are particularly useful in evaluating tissue perfusion. This leads to an increase in spatial resolution, providing more anatomical details in subtle pathologies that can be difficult to visualize on conventional CT, such as the tissue changes involved in acute cholecystitis [29]. Used in both DECT and PCCT, areas of non-enhancement on iodine maps can indicate gallbladder wall hypoperfusion or necrosis, facilitating earlier and more accurate identification of complications such as gangrene or perforation in EC [28,30].

### 6.2. AI

Recent advancements in AI have shown promising potential in enhancing the rapid diagnosis of critical abdominal conditions, such as EC, using CT data. AI-driven algorithms and machine learning, particularly those utilizing deep learning techniques that do not require identification of image features, have been trained on extensive datasets to recognize complex imaging patterns [31,32]. These tools can be trained to identify key radiological features of EC, including pneumatosis, intraluminal gas, and tissue necrosis, which are crucial for timely and accurate diagnosis.

Deep learning models, such as convolutional neural networks that are applicable to radiology, have been at the forefront of this innovation and utilized for image classification [33]. They are capable of processing vast amounts of imaging data to detect subtle abnormalities that might be overlooked by the human eye, thereby enhancing diagnostic precision.

The integration of AI into clinical practice, particularly in emergency departments, holds the potential to significantly reduce diagnostic delays. By providing rapid, automated analysis of CT scans, these AI tools can address chronic radiologist shortage in underserved regions and serve as valuable decision-support systems for radiologists and surgeons, facilitating quicker intervention and potentially improving patient outcomes [34,35,36], especially those with acute conditions. Moreover, AI-driven systems can help standardize interpretations across different healthcare settings, ensuring consistent and reliable diagnoses.

AI is often utilized in day-to-day operations of radiology practices [35]. Artificial intelligence (AI) applications specific to emphysematous cholecystitis (EC) remain limited due to the rarity of the disease; however, advances in AI-based gallbladder imaging for acute cholecystitis provide a relevant framework for future EC-focused development. Deep learning models applied to abdominal CT have demonstrated high accuracy for detecting acute cholecystitis, identifying severe inflammatory features, and predicting suppurative progression by learning imaging patterns such as gallbladder wall thickening, intraluminal abnormalities, and pericholecystic changes—findings that overlap with the imaging spectrum of EC. For example, in one study involving 641 patients at 3 hospitals led by Chen et al., deep learning models using non contrast CT imaging were able to achieve up to 89.81% diagnostic accuracy for acute cholecystitis and up to 85.6% diagnostic accuracy for predicting acute suppurative cholecystitis in patients [37]. This exceeded the performance of radiologist assessments and radiomics models [37]. The results show promise for patients with various types of cholecystitis, given similar imaging findings, such as EC.

In another research study led by Yang et al. using ultrasound images from 250 patients with acute cholecystitis and 270 controls, U-Net artificial intelligence algorithms and VGG-16 architecture were used to detect cholecystitis [38]. The developed ensemble model achieved an overall 86.7% accuracy in detecting acute cholecystitis on ultrasound imaging [38]. For patients requiring cholecystectomy, the AI tool can serve as a tool in detecting cholecystitis patients needing emergent surgery, improving outcomes based on the imaging findings, which are similar for cases with EC.

Automated gallbladder segmentation and region-of-interest localization further support rapid triage and standardized assessment in emergency settings, where early recognition of EC is critical given its high morbidity and mortality. In the future, more prospective multicenter validation using EC-enriched datasets is therefore essential before clinical deployment for this high-risk entity.

The potential impact of AI on the management of acute abdominal conditions, such as EC, is substantial. For example, in one study, a machine learning model was developed using a Shapley Additive explanations algorithm as a predictive tool to anticipate cases of gangrenous cholecystitis using imaging data and to assist physicians in making surgical interventional decisions [39]. In another study led by Zhou et al., a machine learning predictive model using the Random Forest method was developed based on imaging for diagnosing and preoperatively differentiating between xanthogranulomatous cholecystitis and gallbladder cancer [40]. With CT and MRI, the Random Forest model achieved an average accuracy of 90.6%, aiding clinical decision-making [40]. AI technology and applications in imaging and the emergent surgery setting can assist in abdominal pain triage, for example, in patients with acute gallbladder conditions, leading to more timely detection and intervention [41]. Thus, these advances in imaging show promise to transform the diagnostic and treatment landscape of EC, emphasizing a shift towards proactive and precision-based management.

## 7. Conclusions

Emphysematous cholecystitis represents one of the most aggressive manifestations of gallbladder inflammation, arising from the synergistic interaction of ischemic injury, gas-forming bacterial infection, and host vulnerability. The disease underscores a critical principle in abdominal emergencies: pathophysiology alone does not dictate outcome—timeliness of recognition does. In EC, rapid progression toward gangrene, perforation, and septic shock is not merely a consequence of microbial virulence but reflects delayed detection in a condition whose early clinical features may be deceptively nonspecific. The disproportionately high mortality associated with EC highlights the need to view it not simply as a variant of acute cholecystitis, but as a distinct ischemic–infectious entity requiring heightened diagnostic vigilance.

Imaging serves not only as a diagnostic tool but as a determinant of therapeutic trajectory. While CT remains the most sensitive and specific modality for identifying intramural and intraluminal gas and assessing complications, its broader value lies in risk stratification—differentiating uncomplicated infection from necrotizing disease that mandates urgent intervention. Ultrasound and plain radiography continue to provide critical frontline assessment, particularly in unstable or resource-limited environments, yet their susceptibility to diagnostic pitfalls reinforces the importance of multimodality confirmation. Advanced MRI techniques, including diffusion-weighted imaging and MR cholangiopancreatography, extend diagnostic capability in select populations and offer complementary evaluation of adjacent biliary pathology.

Emerging technologies are reshaping the diagnostic framework of emphysematous cholecystitis by enhancing both image quality and clinical decision support. DECT enables material decomposition and improved contrast resolution, allowing more precise differentiation between intramural gas, calcification, hemorrhage, and adjacent biliary structures. This capability is particularly valuable in cases where ultrasound findings are equivocal or when distinguishing emphysematous change from mimics such as porcelain gallbladder or pneumobilia. Photon-counting CT further advances this paradigm by improving spatial resolution and signal-to-noise ratio while simultaneously reducing radiation exposure. Its ability to generate high-resolution spectral data enhances detection of subtle gas locules and early ischemic wall changes that may not yet be radiographically conspicuous on conventional CT. Collectively, these innovations improve diagnostic confidence, refine anatomic delineation for surgical planning, and may allow earlier identification of complications such as microperforation or evolving necrosis.

Parallel to these advancements, AI-assisted image analysis introduces a transformative layer of computational interpretation. Machine learning algorithms trained on large imaging datasets can identify subtle patterns of intramural gas, quantify wall thickening, and detect pericholecystic inflammatory changes with increasing accuracy. Beyond detection, AI offers the potential for automated triage systems that prioritize high-risk imaging studies in emergency workflows, thereby shortening time to diagnosis and intervention. Integration of quantitative imaging biomarkers—such as gas volume, perfusion deficits, or textural heterogeneity—with laboratory indices and comorbidity profiles could support predictive modeling and severity scoring systems tailored to individual patients. Such multimodal data synthesis moves the field toward precision medicine, where imaging not only confirms diagnosis but actively informs risk stratification, therapeutic selection, and prognostication. In this evolving landscape, emphysematous cholecystitis serves as a compelling example of how advanced imaging technologies may transition acute care radiology from reactive confirmation to proactive, data-driven clinical guidance.

Ultimately, the management of emphysematous cholecystitis demands more than technical diagnostic accuracy; it requires interdisciplinary coordination, rapid decision-making, and awareness of imaging limitations and mimics. By synthesizing established radiologic principles with emerging technologies and critical diagnostic considerations, this review frames EC as both a clinical emergency and a model for precision imaging in acute care. Early recognition, informed by evolving imaging capabilities and integrated clinical assessment, remains the cornerstone for reducing morbidity and mortality for patients with emphysematous cholecystitis.

## Data Availability

No new data were created or analyzed in this study.
